# Orthopaedic fracture surgery in polytraumatized patients while on extracorporeal membrane oxygenation (ECMO): A report of two cases

**DOI:** 10.1016/j.tcr.2024.101020

**Published:** 2024-04-08

**Authors:** Jaquelyn Kakalecik, Amanda M. Frantz, Michael T. Talerico, Thomas A. Krupko, Jennifer E. Hagen, Matthew R. Patrick

**Affiliations:** a Department of Orthopaedic Surgery & Sports Medicine, University of Florida, Gainesville, FL, USA; b Department of Anesthesiology, University of Florida, Gainesville, FL, USA

**Keywords:** Polytrauma, Early appropriate care, Extracorporeal membrane oxygenation, ECMO, Orthopaedic surgery, Pelvis fracture, Femur fracture

## Abstract

Extracorporeal membrane oxygenation (ECMO) has become a salvage therapy for patients with severe acute respiratory distress syndrome (ARDS). The management of orthopaedic trauma in ECMO-supported patients with ARDS remains an evolving area of interest. Orthopaedic injuries are often temporized with external fixators, skeletal traction, or splints due to hemodynamic instability as well as concerns of exacerbating underlying pulmonary injury. However, patients requiring ECMO support do not rely on their pulmonary system for oxygenation, the need for delayed fixation may not apply. However, patients utilizing ECMO therapy can have external cardiac and pulmonary support depending on their cannulation strategy, bypassing the need for delayed fixation. We present a case series of two polytrauma patients with ARDS who underwent surgical management of pelvic ring and femoral shaft fractures while receiving ECMO support. Both patients underwent surgical management without complication and were able to be weaned from ECMO and ventilator support postoperatively. These cases highlight the potential benefits to orthopaedic fixation and underscore the need for further clinical research.

## Introduction

Extracorporeal membrane oxygenation (ECMO) is a mechanical circulatory support device employed for patients with cardiogenic shock or refractory hypoxemia. ECMO was first described for treatment of respiratory failure in premature neonates and lung transplant recipients [1,2]. More recently, ECMO has been utilized to treat traumatized patients with severe acute respiratory distress syndrome (ARDS) until their cardiopulmonary insufficiency improves [1,[3], [4], [5], [6]]. The ECMO circuit can be placed in either veno-venous (VV) or veno-arterial (VA) configuration with the cannulations placed in either peripheral or central vasculature. VV ECMO removes deoxygenated blood, oxygenates the blood, removes excess carbon dioxide, and returns the oxygenated blood back to the right atrium when the right internal jugular is used for venous return [2,7]. In addition to ventilatory support, VA ECMO offers circulatory assistance to trauma patients in cardiogenic shock [1,2,5]. VA ECMO is typically reserved for patients with diminished cardiac function or concomitant cardiogenic shock resistant to resuscitation and vasopressor support.

Polytrauma patients develop a massive cytokine inflammatory response which may ultimately lead to the development of ARDS [4]. Apart from the systemic inflammatory response, polytrauma patients with orthopaedic injuries frequently necessitate multiple blood transfusions and are high risk for fat emboli, further increasing the likelihood of developing ARDS [4,8,9]. Early fixation of long bone fractures has been shown to reduce the number of days in the intensive care unit, length of hospital stay, rates of ARDS, pneumonia, and morality rate [[9], [10], [11]]. However, there is also concern that fixation of long bone fractures, particularly with intramedullary implants, poses an additional insult to patients with a severe pulmonary injury [12]. Surgeons must weigh the potential benefits of fixation against the potential exacerbation of a severe pulmonary injury and apply damage control orthopaedic (DCO) principles and stabilize the injury using external fixators, skeletal traction, or splints until the patient's clinical status improves. However, the concept of delayed fixation may not hold true for patients on ECMO. There is little existing literature on the optimal timing or management of orthopaedic trauma patients on ECMO for ARDS. There are four known case reports of patients with femur fractures treated with intramedullary nails while on ECMO [7,8,12,13]. In all cases, the patients recovered uneventfully, were decannulated from ECMO, and discharged from the hospital.

This paper presents the cases of two polytrauma patients with ARDS managed with ECMO. Both individuals underwent surgical management of pelvic ring and femoral shaft fractures while simultaneously receiving ECMO support. Both patients were informed that data concerning the case would be submitted for publication and provided consent. Furthermore, we will offer an overview of ECMO and examine the potential advantages and disadvantages of performing orthopaedic trauma surgery in conjunction with ECMO.

## Case report

### Case 1

**Fig. 1:**
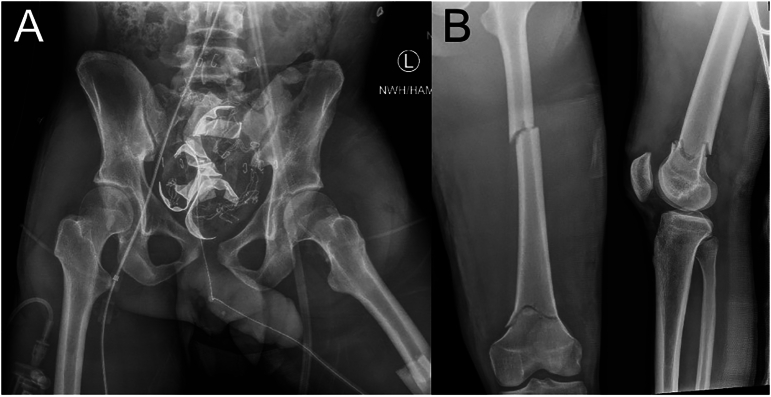
Initial AP pelvis (left), AP left femur (middle), and lateral left femur (right) for case 1. Note the segmental left femur fracture as well as the symphyseal widening with disruption of the left sacroiliac joint.

A twenty-year-old male was involved in a helmeted motorcycle collision. He was intubated on arrival for respiratory distress and hemodynamic instability. The initial trauma survey revealed right pneumothorax, left pneumothorax, hemoperitoneum, liver laceration, renal hematomas, small intracranial hemorrhage, a closed anterior-posterior compression type-2 (APC-2) pelvic ring fracture (Fig. 1A) with injury to the obturator and pudendal arteries, closed right comminuted humeral shaft fracture, and a closed left segmental femoral shaft fracture (Fig. 1B). A pelvic binder was applied and left proximal tibia skeletal traction was placed in the trauma bay.

He was taken emergently to the operative suite for exploratory laparotomy with abdominal and pelvic packing, followed by an angiogram with embolization of the left internal iliac artery and branches of bilateral renal arteries. The patient's respiratory status continued to worsen despite aggressive medical and ventilatory resuscitation and he was urgently placed on VV ECMO by the cardiothoracic surgical team. The patient was brought to the intensive care unit (ICU) where he remained on VV ECMO and ventilator support. He returned to the operating room on hospital day two for repeat exploratory laparotomy, removal of packing, and pelvic external fixation using supra-acetabular Schanz pins.

**Fig. 2:**
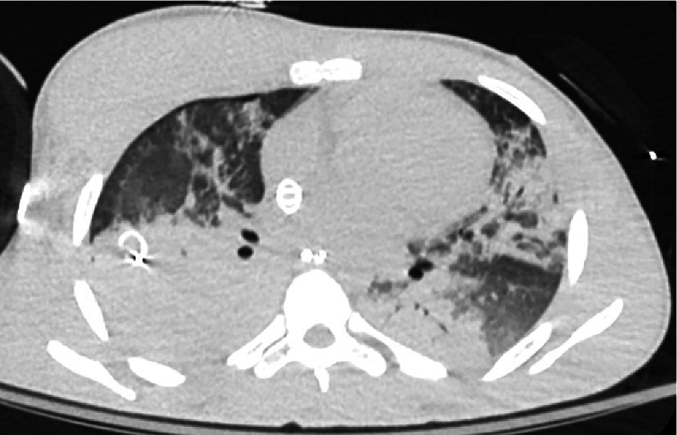
CT chest demonstrating severe pulmonary injury in case 1. Note the extensive bilateral consolidation.

By the third day of hospitalization, he was adequately resuscitated and showed improvement in his oxygenation; however, he still necessitated VV ECMO support. Considering the extent of the pulmonary injury and the ongoing need for resuscitation, the patient would require an extended period of ECMO therapy (Fig. 2). A collaborative discussion amongst the trauma critical care and orthopaedic surgical teams determined the patient would benefit from fixation of his femoral shaft fracture to improve mobilization, prevent pressure injuries, ameliorate pain control, and could allow for prone patient positioning to improve pulmonary therapy. Moreover, fixation of his femoral shaft fracture would decrease bleeding, thereby assisting with ongoing resuscitation efforts. The patient's laboratory values suggested adequate resuscitation (lactate 1.1 mmol/L) and his respiratory status remained stable.

**Fig. 3:**
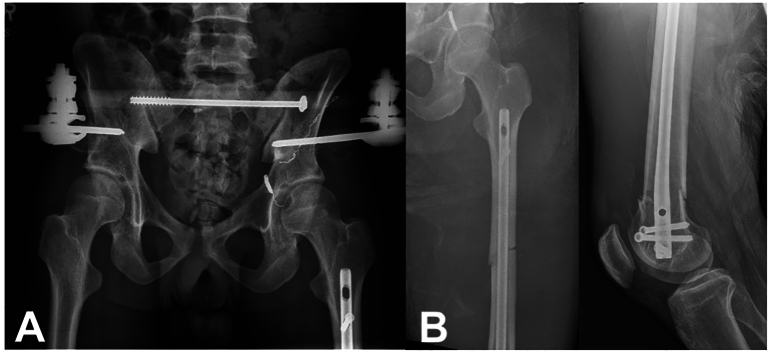
Postoperative AP pelvis (left), AP left femur (middle), and lateral left distal femur (right) for case 1. The pelvic ring injury was treated with anterior pelvic external fixation with percutaneous posterior pelvic ring fixation. The left segmental femur was treated with a retrograde femoral intramedullary nail.

The patient returned to the operative suite on hospital day 3 while on VV ECMO for treatment of the left segmental femoral shaft fracture using a retrograde femoral nail. The patient was positioned supine on the operating room table and a medial parapatellar approach was utilized to insert the nail. The fracture was close reduced, and sequentially reamed from 8.5 mm to 11 mm, increasing by 0.5 mm increments. A 9 mm × 320 mm statically locked nail was utilized for fixation (Fig. 3A). The nail was slightly undersized in diameter and slightly over reamed to minimize the intramedullary pressures during nail insertion, theoretically reducing embolic burden on the lungs. Estimated blood loss was 300 mL during the procedure. The patient tolerated the procedure with no complications. His respiratory status was closely monitored by the anesthesia service and there were no changes in his oxygenation status throughout the procedure. Following fixation of his segmental femur fracture, the general surgery team performed an exploratory laparotomy and fascial closure.

The patient had a tracheostomy placed on hospital day 5. On hospital day 6, the patient became febrile and was discovered to have pneumonia secondary to *Serratia marcescens*, which improved with IV cefepime. On hospital day 8, the patient returned to the operative suite for definitive fixation of his pelvic ring, still reliant on VV ECMO. His left sacroiliac injury was treated with closed reduction and percutaneous placement of a partially-threaded 7.3 mm cannulated transiliac-transsacral style screw with a washer. During manipulation of the pelvis during reduction, moderate bleeding was noted from the left external fixator site. The patient's abdomen was tense to palpation with increasing bladder pressures concerning for abdominal compartment syndrome. The general surgeons performed an exploratory laparotomy and evacuated a large volume of bloody ascitic fluid with no identifiable active bleeding. Fixation of the anterior pelvic ring was aborted; the pelvis was packed with sponges and the external fixator was secured. Total estimated blood loss was 600 mL. The patient was then transported to the interventional radiology suite for angiography and possible embolization. A terminal branch of the left superior gluteal artery and left hypogastric artery had active extravasation, which were each selectively embolized. The large amount of ascitic fluid and bleeding was likely multifactorial in the setting of the patient's concomitant liver injury, renal dysfunction, and fluid retention while on ECMO.

The patient returned for repeat exploratory laparotomy on hospital day nine, ten, and eleven for sequential abdominal closure. The anterior pelvic ring reduction remained acceptable, and thus the anterior injury was treated definitively with an external fixator given the multiple pelvic packings and prolonged open abdomen increasing the infection risk (Fig. 3B). The patient was decannulated from VV ECMO on hospital day 17 and underwent open reduction and internal fixation of his right humeral shaft fracture on hospital day 21. He was discharged to a long-term acute care facility for ventilatory weaning on hospital day 23 and returned home after approximately 10 days in the facility with no further respiratory needs. His tracheostomy tube was subsequently removed, and his orthopaedic injuries healed uneventfully. By 6 months, he had resumed all activities of daily living and had returned to the work force.

### Case 2

**Fig. 4:**
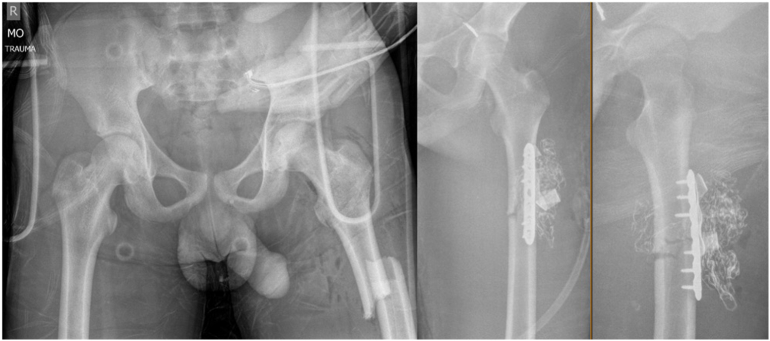
Initial AP pelvis (left), AP left femur (middle), and lateral left femur (right) for case 2. Note the anterior 3.5 mm LCP plate and anterior thigh packing performed as part of initial damage control orthopaedics. The bilateral SI joint dislocations are not well visualized on initial pelvic radiograph.

A seventeen-year-old male was the restrained passenger in a rollover motor vehicle collision. On arrival, the patient was hemodynamically stable with a negative Focused Assessment with Sonography in Trauma (FAST) examination. While obtaining the trauma CT scans, the patient became hemodynamically unstable with pulseless cardiac arrest. He was intubated and his cardiac function restored after one round of advanced cardiac life support. Once stabilized, the CT of his chest, abdomen and pelvis revealed a small splenic hematoma, significant bilateral pulmonary contusions, left superior gluteal artery injury with active extravasation, bilateral sacroiliac (SI) joint dislocations, and a left type 3 A open subtrochanteric femur fracture. He was transported to the operating suite for a combined interventional radiology-orthopaedic procedure. Distal femoral skeletal traction was applied, and the open femur fracture was debrided and irrigated. A considerable venous bleeding was observed from the femur and adjacent soft tissues, none of which was pulsatile, consistent with coagulopathy. A 7-hole 3.5 mm LC-DCP plate was applied to the femur to provide provisional stabilization and minimize blood loss from the medullary canal The traumatic wound was packed with lap sponges soaked with 2 g of tranexamic acid and 5000 IU thrombin to assist with hemostasis and was closed primarily (Fig. 4).

The interventional radiologists performed angiography and selective embolization of the inferior and superior gluteal arteries. Resuscitation in the operating room required a substantial amount of various blood products. Throughout the duration of the case, the patient's pulmonary function continued to worsen with hypoxemia refractory to conventional ventilation. The patient was placed on VV ECMO by the cardiothoracic surgeons. The patient was then transported to the ICU for continued resuscitation and medical management.

A multidisciplinary discussion was held amongst the patient care team on hospital day 3. Considering the severity of his pulmonary contusions, the patient's care team felt he would require prolonged VV ECMO and ventilator support. He would, however, need definitive stabilization of the fracture to perform pulmonary therapy more effectively and assist with ongoing resuscitation. The potential pulmonary repercussions of intramedullary fixation, including fat emboli, pneumonia, and worsening of acute lung injury, would likely be mitigated with VV ECMO. His respiratory status remained stable, and the patient was adequately resuscitated (lactate 1.1 mmol/L). The patient returned to the operating room on hospital day 3 for removal of packing and repeat debridement of the open subtrochanteric femur fracture followed by fixation with an antegrade intramedullary nail. The patient was positioned supine on a radiolucent table, and the fracture was open reduced through the traumatic wound. The previously placed 3.5 mm LC-DCP plate was removed from the femur prior to nail insertion. The canal was sequentially reamed by 0.5 mm increments up to 12 mm utilizing a standard reamer. A size 10 × 340 mm nail was selected (Fig. 6). Similar to case 1, the medullary canal was over-reamed by 2 mm to minimize the intramedullary pressures during nail insertion. The patient tolerated the procedure without changes in oxygenation status or other complications during the case. Estimated blood loss was approximately 200 mL.

Later that evening on hospital day 3, the patient had bilateral chest tubes placed for pleural effusions. Notably, the trauma critical care team did not attribute the requirement for chest tube placement to his recent orthopaedic surgery.

**Fig. 5:**
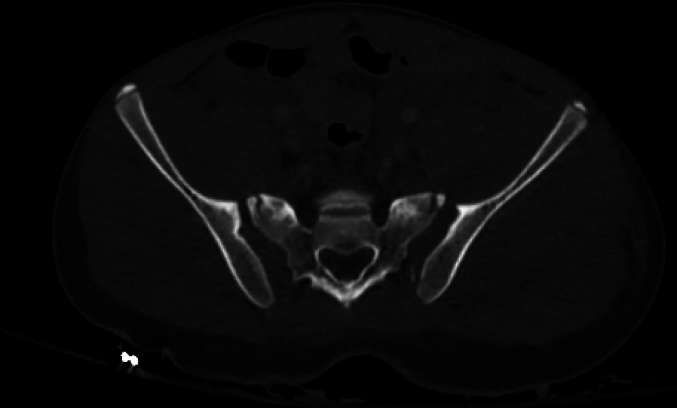
Axial cut from CT pelvis demonstrating bilateral SI joint dislocations in case 2.

**Fig. 6:**
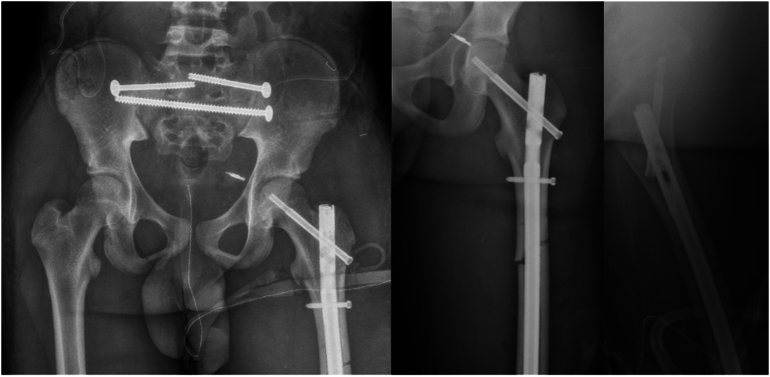
Postoperative AP pelvis (left), AP left femur (middle) and cross-table lateral of left femur (right) for case 2. The subtrochanteric femur fracture was treated with an antegrade reconstruction intramedullary nail. The temporary 3.5 mm LCP stabilization plate was removed. The bilateral SI joint dislocations were treated with open reduction and placement of sacroiliac style screws in the upper sacral segment and a transiliac transsacral screw in the lower sacral segment.

On hospital day 4, another multidisciplinary discussion was held amongst the patient's care team. The patient's respiratory status had improved following placement of bilateral chest tubes, though still reliant on VV ECMO. He was brought back to the operating room suite for fixation of his bilateral sacroiliac joints (Fig. 5). The patient was positioned supine on a radiolucent operating room table. The bilateral SI joints were unable to be close reduced. Both SI joints were open reduced through a lateral window and were each stabilized with a partially-threaded 7.3 mm sacroiliac style screw in the upper sacral segment. An additional fully-threaded 7.3 mm transiliac-transsacral screw was placed in the second sacral segment for additional fixation (Fig. 6). A stress examination was performed on the anterior pelvic ring, which was stable and required no additional fixation. Estimated blood loss was 700 mL, and the patient tolerated the procedure well without complications.

The patient was decannulated form VV ECMO on hospital day 6 and was extubated on hospital day 8. His chest tubes were removed on hospital day 11 and he was discharged home without oxygen requirement on hospital day 13. The patient began weight bearing seven weeks postoperatively and was walking without assistance 3 months after fixation. At his three-month clinic visit, the patient was ambulating without assistance, had bridging callus on femur radiographs, and maintained reduction of his pelvic ring injury. He was subsequently lost to follow-up after his three-month visit.

## Discussion

ARDS is characterized by severe respiratory failure and refractory hypoxemia. Risk factors for ARDS include, but are not limited to, pulmonary contusions, multiple blood transfusions, and systemic inflammatory response syndrome (SIRS), which are often present following severe trauma [3,4]. Severe pulmonary injury can lead to refractory ARDS, necessitating high respiratory rates, airway pressures, and oxygen concentrations, which can perpetuate a cycle of barotrauma and exacerbate the pulmonary injury [1]. The prevalence of traumatic lung injury leading to ARDS remains around 13.4 to 18.1 %, with 25 % of blunt trauma mortalities attributed to blunt chest injury [6]. ECMO has emerged as an adjunct in the management of ARDS, offering a salvage therapy to provide respiratory and circulatory support when conventional ventilation and maximum pharmacologic usage is inadequate [4,8]. ECMO benefits in the trauma population include systemic warming and correcting hypoxia or hypercapnia resulting in acidosis [6].

Patients are placed on ECMO as a bridging therapy in the setting of cardiopulmonary failure to achieve organ recovery, transplant, or decision which can sometimes end with palliation [14]. The majority of patients placed on ECMO are non-traumatic lung injury to allow the organ to recover with a small subset of blunt or penetrating trauma with comorbid acute lung injury. ECMO has three mean configurations including venoarterial (VA), arteriovenous (AV) or venovenous (VV) orientation. VV ECMO removes blood from the venous system, oxygenates and removes carbon dioxide from the blood, and returns it to the venous system, bypassing the pulmonary system [3]. VA ECMO provides cardiac circulatory support while also oxygenating the blood; blood is removed from the venous system and returned to the arterial system [3]. VV ECMO is typically used for resistance hypoxemia, while VA ECMO is used when cardiac function is compromised and hypoxemia persists despite maximum flow on VV ECMO [4]. AV configuration is the least common and known as “artificial lung”, where no pump is utilized in the circuit and the patient's native cardiopulmonary system drives blood from the inflow arterial cannula to the patient's venous circuit with oxygenated blood [14].

The main contraindication with polytrauma patients is the potential need for anticoagulation to prevent clotting in the ECMO circuit. As anticoagulation can worsen bleeding in this high risk population there are reassuring case reports and case series have shown to be ECMO to be efficacious in the adult and pediatric trauma population without increasing mortality from hemorrhage [6,15]. Patients are also at risk for bleeding with subsequent qualitative platelet dysfunction and thrombocytopenia and consumption of coagulation factors in the setting of ECMO use. There are new areas of study assessing using VV ECMO without anticoagulation, which would reduce the increased risk of bleeding in the polytrauma population [6].

Another way to avoid uncontrollable hemorrhage is to utilize a reversible medication like systemic heparin and to reduce the target goal range from activated clotting time 180–200 s or Factor Xa levels 0.35 to 0.7 U/mL, down to 155 to 170 s or 0.25 to 0.3 U/mL respectively for high-risk patients [15]. A case series utilized the above goals on VV and VA ECMO by Skarda et al. noted patients undergoing operative procedures from open reduction and internal fixation of an open humeral fracture to other invasive surgeries including exploratory laparotomy and thoracotomy without ECMO complications [15]. The use of reversal or holding of anticoagulation needs to weigh the complications of bleeding with possible circuit thrombosis, which can lead to patient mortality [14].

While bleeding can be temporized through transfusion and minimizing anticoagulation, the management of ECMO in the perioperative environment is tenuous as the pump is preload dependent and afterload sensitive which can be difficult to manage in the setting of hemorrhagic shock. Specifically, in the orthopaedic surgery population requiring femur, pelvis or hip surgery, normal blood sparing techniques cannot be employed in the operating room, thus high volume donor transfusions are required which can lead to worsening acute lung injury via activation of the immune system [16]. The anesthesia choice in the operating room is limited to total intravenous medications secondary to lung injury that precludes the use of inhalation agents [14]. The vasodilation from anesthetics will cause an increased need for vasopressor support in the perioperative environment which can affect tissue perfusion and healing of intraoperative repairs.

ECMO is also associated with other risks and challenges. ECMO frequently requires anticoagulation with heparin or the use of heparin-coated circuits, which not only increase the risk for bleeding but can subject the patient to heparin-induced thrombocytopenia [3]. There is also the risk for infections of cannula-sites and the possibility of air emboli or thrombi [7]. Specifically, procedures involving the pelvis and proximal thigh preclude the application of a tourniquet, making hemorrhage a substantial and legitimate concern. Chen et al. conducted a study on 810 ECMO patients, 191 of which underwent an orthopaedic surgical procedure [6]. Interestingly, the mortality rate did not significantly differ between those who underwent orthopaedic surgery and those that did not, although there was increased mortality in patients who underwent other types of surgical procedures such as abdominal, brain, or thoracic surgery [6].

The timing of fracture fixation has been extensively studied in the orthopaedic literature [9,[17], [18], [19]]. In the 1980s, early stabilization of long bone fractures within 24–48 h of hospital admission was prioritized to reduce pulmonary complications, allow for early mobilization, and shorter hospital stays [9,17]. Further understanding of the systemic inflammatory response to severe trauma led to a shift in orthopaedic practice. Early fracture surgery, particularly with intramedullary implants, can lead to fat emboli and hypoxic events, which can act as a “second hit” on the cardiopulmonary system [18,19]. Instead, patients are stabilized with splints, skeletal traction, or external fixators until they are hemodynamically stable and who have met resuscitative criteria.

However, surgical timing and management of orthopaedic injuries for patients on ECMO with ARDS remains unclear. It is plausible that the definitive fixation of long bone injuries does not adversely impact the recovery of patients on ECMO. In fact, it might contribute to resuscitative efforts, potentially expediting cardiopulmonary recovery. Prone positioning has been shown to decrease mortality in ARDS due to redistribution of tidal volumes and alterations in the ventilation-perfusion gradient [4,[20], [21], [22]]. Achieving a prone position is challenging when skeletal traction or external fixation is in place. Definitive treatment of orthopaedic injuries facilitates patient positioning, thereby enhancing pulmonary hygiene. Furthermore, stabilizing orthopaedic injuries improves pain management and can reduce the requirement for analgesics, which incur the side effect of respiratory depression [1].

Though the existing literature is limited to case reports, these instances have yielded positive outcomes with patients ultimately recovering and being discharged from the hospital [7,8,12,13]. Bosch et al. reported on an 18-year-old patient with a femoral shaft fracture treated with an antegrade femoral nail while on VV ECMO [12]. The reamer-irrigator-aspirator (RIA) device was utilized to decrease embolic load [12]. The patient was subsequently decannulated from ECMO and discharged from the hospital. Carraro et al. and Chauhan et al. describe treatment of femoral shaft fractures with intramedullary implants and standard reaming while on VV ECMO; both cases resulted in successful ECMO weaning and eventual extubation [7,8]. Calvo et al. published a case report on a 25-year-old female with bilateral traumatic lower extremity amputations and a femoral shaft fracture initially stabilized with an external fixator who subsequently developed refractory respiratory failure requiring VV ECMO [16]. She developed polymicrobial infections at bilateral amputation sites, and her external fixator was removed and converted to an intramedullary implant due to concern for pin site infection [16]. The patient had no perioperative complications and was successfully weaned from ECMO 7 days after femur fixation. Collectively, these reports underscore the evolving approach toward fracture management in ECMO-supported patients and the potential benefits to fixation on patient outcomes.

This case series broadens the scope of fracture fixation in patients with orthopaedic injuries treated while on ECMO for ARDS. Previous case reports predominantly focused on the treatment of femur fractures [7,8,12,13]. This case series includes two patients who also underwent fixation of pelvic ring injuries, shedding light on a previously underrepresented aspect of acute orthopaedic care. As ECMO becomes increasingly prevalent and accessible in healthcare systems, it will likely become a more common clinical challenge for orthopaedic providers. Both patients included in this study recovered from their orthopaedic procedures without complications, were decannulated from ECMO shortly after surgery, resumed ambulation, and had no oxygen requirement at their most recent orthopaedic follow-up. This case series demonstrates that definitive fixation of long bone fractures and pelvic ring injuries can be achieved successfully and without delay in patients on ECMO for ARDS, which can help establish guidelines for acute orthopaedic surgical management of polytrauma patients requiring ECMO support.

## Conclusion

This paper presents two polytrauma patients with severe ARDS who underwent surgical management of pelvic ring and femoral shaft fractures while receiving VV ECMO support. Despite concerns for potential additional pulmonary insult, the patients in this study experienced successful outcomes and were able to be weaned from ECMO and ventilatory support. As ECMO continues to become more available in healthcare systems, orthopaedic providers must be familiar with the risks and benefits to treating of polytrauma patients while on VV or VA ECMO. This case series underscores a potential role of definitive treatment instead of temporizing measures for patients requiring ECMO support and highlights an area for large scale research efforts.

## Institutional Review Board (IRB)

UF IRB # IRB202202009.

## Funding

This research did not receive any specific grant from funding agencies in the public, commercial, or not-for-profit sectors.

## CRediT authorship contribution statement

**Jaquelyn Kakalecik:** Conceptualization, Data curation, Methodology, Writing – original draft, Writing – review & editing. **Amanda M. Frantz:** Writing – review & editing. **Michael T. Talerico:** Writing – review & editing. **Thomas A. Krupko:** Writing – review & editing. **Jennifer E. Hagen:** Writing – review & editing. **Matthew R. Patrick:** Conceptualization, Methodology, Writing – review & editing.

## Declaration of competing interest

The authors of this paper have no financial disclosures.
